# Melkersson-Rosenthal syndrome in children and adolescents: a series of seven cases^☆^

**DOI:** 10.1016/j.abd.2021.05.020

**Published:** 2022-07-12

**Authors:** Camila Fátima Biancardi Gavioli, Yasmin da Silva Amorim Cidade, Giovanna Piacenza Florezi, Silvia Vanessa Lourenço, Marcello Menta Simonsen Nico

**Affiliations:** a Department of Dermatology, Faculty of Medicine, Universidade de São Paulo, São Paulo, SP, Brazil; b Department of Pathology, Faculty of Odontology, Universidade de São Paulo, São Paulo, SP, Brazil

Dear editor,

Melkersson-Rosenthal syndrome (MRS)/orofacial granulomatosis is characterized by the triad of recurrent orofacial edema, recurrent peripheral facial palsy, and fissured tongue.^1^ The triad is found in 8% to 45% of cases; most patients present with the oligosymptomatic or monosymptomatic forms of the disease.^1^ The most common clinical manifestation is lip swelling (granulomatous cheilitis).^1^ The disease mainly affects young adults; pediatric cases are rarely described. A recent series described three cases and reviewed 116 previously published ones.^2^

The cause of MRS is unknown. The authors of the present study demonstrated an increase in the expression of HLA A*02, HLA DRB1*11 to HLA DQB1*03 and a decrease in the levels of HLA A*01, HLA DRB1*04, HLA DRB1*07, and HLADQB1*02 in patients with MRS when compared to the control group, indicating genes that may predispose to or protect against the disease.^3^ An association between MRS and Crohn's disease (CD) has been reported by some authors.^4^

The histopathological findings of MRS include non-caseating granulomas similar to CD, which may suggest that MRS and CD might be part of the same clinicopathological spectrum.^1^, ^4^

The present report describes seven cases of MRS in children and adolescents, drawing attention to a possible association with CD.

The analyzed data from the cases diagnosed with granulomatous cheilitis/MRS/orofacial granulomatosis were: sex, age, lesion location, neurological impairment, and colonoscopy exams (Figure 1, Figure 2, Figure 3, Figure 4, Figure 5, Figure 6, Figure 7 and Table 1). The diagnosis was confirmed by the histopathological analysis.^5^

**Figure 1:**
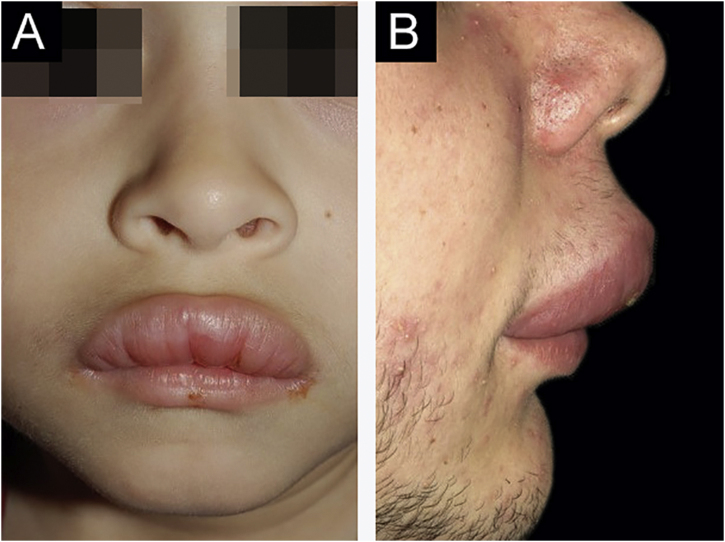
Clinical characteristics of cases 1 and 2. (A), Case 1 ‒ macrocheilia. (B), Case 2 ‒ macrocheilia.

**Figure 2:**
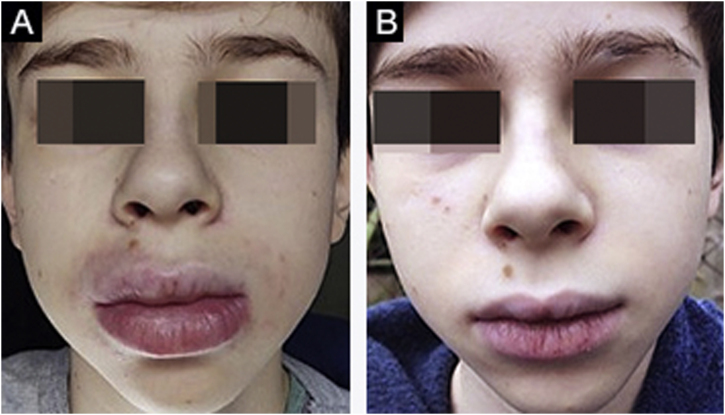
(A), Case 3 ‒ macrocheilia. (B), Same patient during treatment with dapsone: good response.

**Figure 3:**
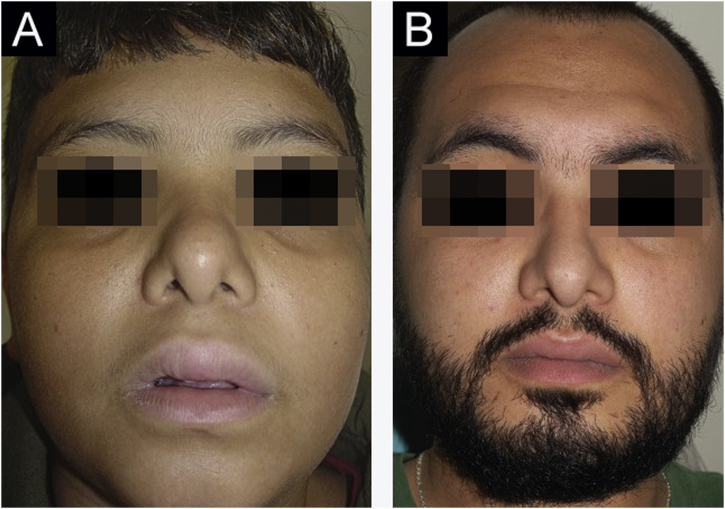
(A), Case 4 ‒ diffuse facial edema. (B), Same patient, as an adult ‒ complete resolution of edema.

**Figure 4:**
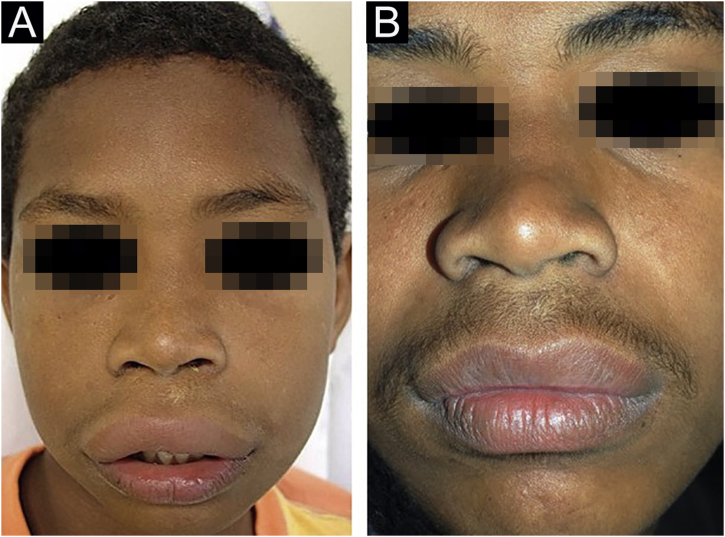
(A), Case 5 ‒ macrocheilia. (B), Same patient, as an adult after several surgical procedures.

**Figure 5:**
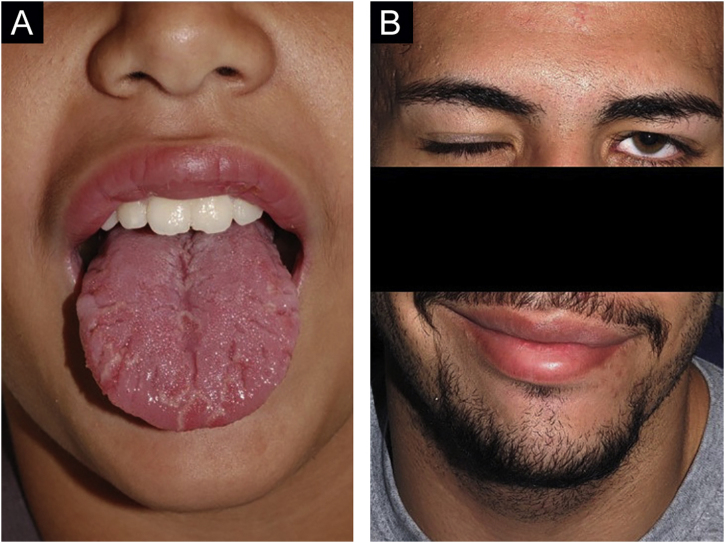
(A), Case 6 ‒ macrocheilia, geographic and fissured tongue. (B), Same patient at age 19 during a bout of facial palsy; the macrocheilia had already disappeared.

**Figure 6:**
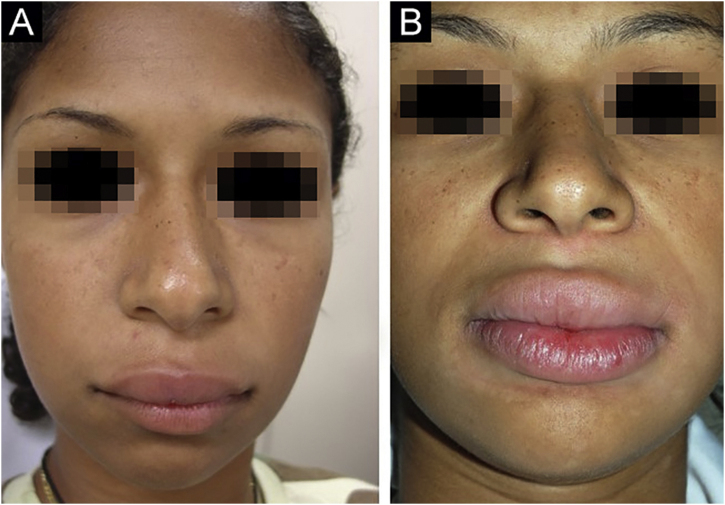
(A), Patient 7 ‒ macrocheilia associated with mild facial edema. (B), Same patient during a severe crisis that showed to be resistant to the therapies.

**Figure 7:**
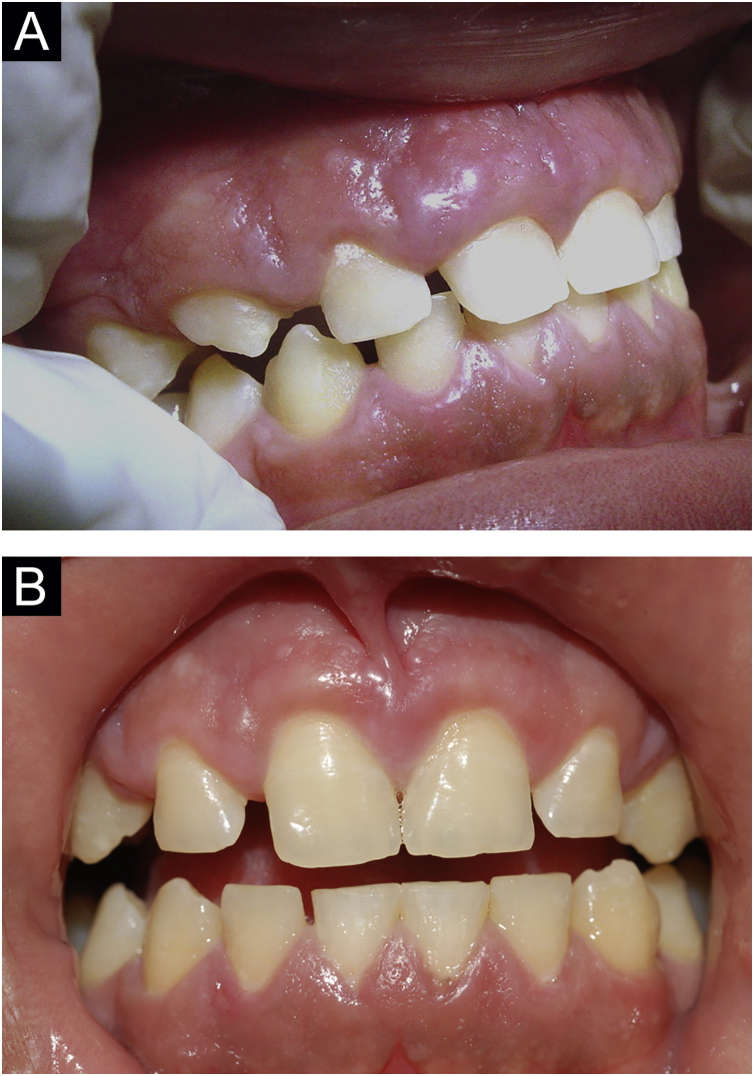
(A), Patient 4 ‒ granulomatous gingivitis.^10^ (B), Same patient after gingivoplasty.

**Table 1:** Characteristics of the seven patients with pediatric MRS.

Case/ Sex/ Ethnicity	Age at symptom onset	Age at diagnosis	Affected site	Intraoral involvement	Tongue involvement	Facial palsy	Systemic involvement	Treatment	Follow-up
1/M/C	30 m	43 m	Upper lip	No	No	No	Normal colonoscopy	Thalidomide, 50 mg/day, good response	2 y
2/M/C	14 y	15 y	Upper lip	No	No	No	Normal colonoscopy	Dapsone, 100 mg/day	Lost to follow-up
3/M/C	11 y	12 y	Upper and lower lip	No	No	No	Normal colonoscopy	Dapsone, 100 mg/day, good response	9 m
4/M/C	10 y	11 y	Lips and face	Granulomatous gingivitis	Geographic tongue	No	Colonoscopy- Crohn’s disease at 21 years	Dapsone, 100 mg/day (failure)	14 y
								Thalidomide, 100 mg/day (failure)	
								Azathioprine, 150 mg/day (good response) gingivoplasty	
5/M/AB	8 y	9 y	Upper and lower lip	No	Fissured tongue	No	Non-epileptic seizures	Thalidomide, 100‒200 mg/day (poor result)	
							Normal colonoscopy	Intralesional Triamcinolone (failure), three cheiloplasties	
6/M/C	11 y	12 y		Granulomatous gingivitis	Geographic and fissured tongue	Yes	Colonoscopy- Crohn’s disease at 17 years	Thalidomide, 100 mg/day, good response	6 y
7/F/MR	15 y	16 y	Lips and face	Granulomatous gingivitis and palatitis	Fissured tongue	No	Colonoscopy not performed	Dapsone, 100 mg/day	4 y
								Prednisone, 40 mg/day	
								Intralesional Triamcinolone (all failed)	

M, Male; F, Female; m, months; y, years; C, Caucasian; AB, African-Brazilian; MR, Mixed-race.

This sample represents the largest case series of children with MRS in Latin America.^6^ The authors’ oral mucosa diseases group conducts 900 medical consultations per year and, over a period of 20 years, only five pediatric MRS cases were seen (Cases 1 and 4 through 7); Cases 2 and 3 came from the private practice of one of the authors.

There was a predominance of male patients; the only female patient noticed symptoms at age 15. The cases reviewed by Savasta et al. showed a prevalence of female patients.^2^ Patient 1 is one of the youngest subjects ever reported (Fig. 1A).

Only patient 6 reported a previous bout of facial palsy, having also had an episode observed by the authors. Facial palsy affected 61/116 (52.6%) of the previously reported pediatric cases.^2^ Patient 5 had seizures of unknown cause; the neurology team associated them with MRS.^1^

Cases 4, 5, 6 and 7 had fissured tongue; cases 4 and 6 had geographic tongue (Fig. 5A; Case 6 had concomitant fissured tongue). Geographic tongue is characterized by the presence of migrating areas of depapilation surrounded by a serpiginous edge. Histopathology is identical to that of psoriasis and is currently considered a mucosal manifestation of the latter. There is a significant association between psoriasis and Crohn's disease.^7^ A fissured tongue usually occurs in cases of persistent geographic tongue; the two findings often coexist and represent the same process at different stages.^8^

Cases 3 and 6 were diagnosed with CD, which was detected many years after the diagnosis of MRS, after controlling for orofacial symptoms. The routine investigation of MRS at the authors’ service currently includes periodic colonoscopy exams.

An association between MRS and CD has been reported.^1^ The authors’ group published the results of a study with HLA in 36 adult and pediatric cases^3^ and confirmed the association between MRS and CD in five cases (all had MRS HLA alleles and three had DC alleles). The genetic results obtained by the authors suggest that MRS and CD are distinct diseases, but they may be associated.

No treatment is effective for all cases of MRS; there are no controlled studies. There are no clinical elements that indicate the choice of a particular drug. It is believed that the selected drug should be used for at least three months before being considered a failure, as the response is slow. The authors’ preferred drug is thalidomide, based on their experience with adult patients.^9^ Five patients received thalidomide and two received dapsone. Only one patient (Case 6) showed significant improvement with thalidomide. Patient 1 is slowly improving after one year of follow-up. Cases 4 and 5 only improved when they became adults, despite treatment with different drugs. It is not known whether the improvement occurred due to the last drug used or if it occurred spontaneously. Case 3 showed a good response to dapsone; some subsequent recurrences were controlled with short cycles of oral corticosteroids (Fig. 2A‒B). Case 4, after several attempts, only improved with azathioprine and gingivoplasty (Fig. 3A‒B). Case 5 only improved with surgical treatment (Fig. 4A‒B). Patient 7 was refractory to all therapies and, after several years and crises, was lost to follow-up (Fig. 6A‒B). The authors’ group does not use immunobiologicals, although there are grounds for their use.^9^, ^10^, ^11^

Surgical treatment is an option for localized and refractory disease. Three cheiloplasties were performed in Case 5.

Cases 4, 6, and 7 had granulomatous gingival infiltration that has been rarely reported.^12^ This manifestation should be actively looked for and treated, as it can lead to periodontal involvement. These lesions are resistant to pharmacological treatment; gingivoplasty is an excellent therapeutic option (Fig. 7).

In conclusion, with the exception of Case 2, all the others presented herein were followed for several years, highlighting the variability of their evolution and the difficulty in managing this disease.

## Financial support

FAPESP ‒ (2017/26990-8) and FUNADERSP ‒ (29/2016).

## Authors' contributions

Camila Fátima Biancardi Gavioli: Statistical analysis; approval of the final version of the manuscript; design and planning of the study; drafting and editing of the manuscript; collection, analysis, and interpretation of data; effective participation in research orientation; intellectual participation in the propaedeutic and/or therapeutic conduct of the studied cases; critical review of the literature; critical review of the manuscript.

Yasmin da Silva Amorim City: Statistical analysis; approval of the final version of the manuscript; drafting and editing of the manuscript; collection, analysis, and interpretation of data; effective participation in research orientation; critical review of the literature.

Giovanna Piacenza Florezi: Design and planning of the study; collection, analysis, and interpretation of data; intellectual participation in the propaedeutic and/or therapeutic conduct of the studied cases; critical review of the literature.

Silvia Vanessa Lourenço: Statistical analysis; approval of the final version of the manuscript; design and planning of the study; drafting and editing of the manuscript; collection, analysis, and interpretation of data; effective participation in research orientation; intellectual participation in the propaedeutic and/or therapeutic conduct of the studied cases; critical review of the literature; critical review of the manuscript.

Marcello Menta Simonsen Nico: Statistical analysis; approval of the final version of the manuscript; design and planning of the study; drafting and editing of the manuscript; collection, analysis, and interpretation of data; effective participation in research orientation; intellectual participation in the propaedeutic and/or therapeutic conduct of the studied cases; critical review of the literature; critical review of the manuscript.

## Conflicts of interest

None declared.
